# Access to and application of recanalizing therapies for severe acute ischemic stroke caused by large vessel occlusion

**DOI:** 10.1186/s42466-023-00245-9

**Published:** 2023-05-18

**Authors:** Julian Bösel, Gordian J. Hubert, Jessica Jesser, Markus A. Möhlenbruch, Peter A. Ringleb

**Affiliations:** 1 Department of Neurology, University Hospital Heidelberg, Heidelberg, Germany; 2 TEMPiS Telestroke Center, Department of Neurology, München Klinik, Academic Teaching Hospital of the Ludwig-Maximilians-University, Munich, Munich, Germany; 3 Department of Neuroradiology, University Hospital Heidelberg, Heidelberg, Germany

**Keywords:** Large vessel occlusion, Severe stroke, Acute ischemic stroke, Intravenous thrombolysis, Endovascular stroke treatment, Stroke thrombectomy

## Abstract

**Background:**

Groundbreaking study results since 2014 have dramatically changed the therapeutic options in acute therapy for severe ischemic stroke caused by large vessel occlusion (LVO). The scientifically proven advances in stroke imaging and thrombectomy techniques have allowed to offer the optimal version or combination of best medical and interventional therapy to the selected patient, yielding favorable or even excellent clinical outcomes within time windows unheard of before. The provision of the best possible individual therapy has become a guideline-based gold standard, but remains a great challenge. With geographic, regional, cultural, economic and resource differences worldwide, optimal local solutions have to be strived for.

**Aim:**

This standard operation procedure (SOP) is aimed to give a suggestion of how to give patients access to and apply modern recanalizing therapy for acute ischemic stroke caused by LVO.

**Method:**

The SOP was developed based on current guidelines, the evidence from the most recent trials and the experience of authors who have been involved in the above-named development at different levels.

**Results:**

This SOP is meant to be a comprehensive, yet not too detailed template to allow for freedom in local adaption. It comprises all relevant stages in providing care to the patient with severe ischemic stroke such as suspicion and alarm, prehospital acute measures, recognition and grading, transport, emergency room workup, selective cerebral imaging, differential treatment by recanalizing therapies (intravenous thrombolysis, endovascular stroke treatmet, or combined), complications, stroke unit and neurocritical care.

**Conclusions:**

The challenge of giving patients access to and applying recanalizing therapies in severe ischemic stroke may be facilitated by a systematic, SOP-based approach adapted to local settings.

## Introduction

Patients with stroke symptoms will most likely be brought to the attention of medical services by the alarming call of family, friends, bystanders, a consulted medical practitioner or, less likely, the patient himself. Emergency medical service (EMS) providers will then often be the first professionals to see the patient and have to confirm or reject suspicion of stroke on clinical grounds and grade that stroke. Unless imaging can be done on the spot [as by mobile stroke unit (MSU) ambulances], it will remain uncertain whether the stroke is caused by ischemia [80%, acute ischemic stroke (AIS)] or hemorrhage [intracerebral hemorrhage (ICH)]. It is possible, however, to confidently grade the stroke severity. In the primary hospital, the patient has to receive an adequate emergency room (ER) workup and rapid cerebral imaging, including examination of the cerebral vessels. This will determine, among other factors, whether and what kind of recanalizing treatment is acutely indicated. This may lead to a secondary transfer, if that primary hospital is not capable of endovascular stroke treatment (EVT). The transfer may be preceded by the start of intravenous thrombolysis (IVT). Depending on the clinical, radiological, and local scenario, as well as the time from stroke onset, the patient may receive thrombectomy, a complex catheter-based therapy involving not only an interventional neuroradiologist and angiography assistant, but also a multidisciplinary team taking care of sedation, airway and possibly ventilation, blood pressure, etc. before, during and after the procedure. Thereafter, depending on the success of the recanalizing therapies, the clinical state of the patient and other peri**-**/postinterventional factors, the patient needs to be transferred to and further treated on a stroke unit (SU) or a (neuro) critical care unit (NCCU). This chain of action must be applied as rapidly as possible. The following is for the most part based on current guidelines of acute therapy for AIS such as those from Germany [1], USA [2], and the European Stroke Organization (ESO) [3, 4].

### Definition

Endovascular stroke therapy (EVT): Whenever EVT is used in the following, it is meant to be synominous with mechanical thrombectomy, i.e. removal of a brain vessel—obstructing clot by stent retrieval, aspiration or other techniques via a catheter. The broader term intra-arterial stroke therapy (IAT) may stand for or include the application of thrombolytics intra-arterially via a catheter, but its evidence is at no means comparable to that of mechanical thrombectomy. If IAT is addressed, this is specifically clarified in the following comments. To date, IAT is in most centers regarded a rescue therapy in case mechanical thrombectomy fails.

### Flow chart

**Fig. 1:**
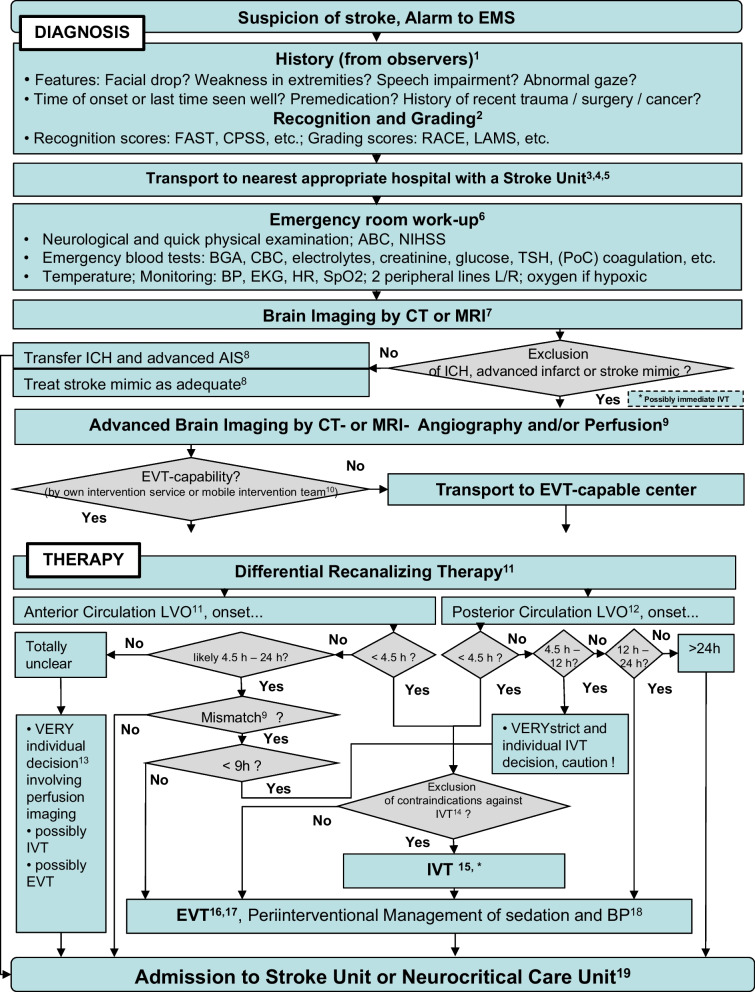
SOP Recanalizing therapy for large vessel occlusion acute ischemic stroke

The steps of this suggested standard operating procedure can be found in Fig. 1. The following text addresses footnotes within the figure.

### Comments on flow chart

1. When taking the history from either the patient or the observers, the following items are particularly important to ask for or take in over the phone or by the EMS/emergency physician on the scene: Clinical syndrome (especially paresis, speech-/language disturbance, gaze abnormality), time and acuity of onset or last time seen well, premedication (particularly anticoagulation), past medical history with focus on allergies to contrast agents and alteplase contraindications (recent trauma, stroke, surgery, cancer, coagulopathy), prestroke general condition (status of dependency, etc.), and contact number of next of kin for further questions.
2. Recognition of the stroke as opposed to other medical conditions, i.e. establishing justified suspicion of stroke, is the first important step by those professionals making the first contact. Several clinical tools (scores) have been investigated for stroke recognition by the EMS. A Cochrane analysis from 2019 suggested that the Cincinnati Prehospital Stroke Scale (CPSS) had the highest sensitivity when performed in the ambulance, but other scores are also in widespread use [5]. Another common and validated recognition score recommended in guidelines [1] is the Face-Arm-Speech-Time (FAST) test.
3. Prehospital workup should not be different in patients with severe or mild to moderate stroke syndrome as the latter stroke patients may also be eligible for recanalizing therapies. The overall concept must be to diagnose and process the patient as fast as possible to the closest stroke unit. All patients should receive a quick standard EMS routine check (such as ABCDE). In some patients, vital functions are compromised, e.g. due to loss of airway protective reflexes and aspiration, decline in level of consciousness in basilar artery occlusion, hypertensive crisis, or acute comorbidities such as myocardial infarction. In these cases an emergency physician must be called onto the scene to provide life supporting measures if these cannot be provided by the EMS paramedics (national differences). The EMS has to inform the target hospital of the arrival of a patient with suspected stroke and of the estimated time of arrival.
4. To differentiate between stroke patients with LVO and those without, many scales have been studied. An observational study from the Netherlands (PRESTO) compared eight different scales and found that some scales can detect anterior LVO with acceptable to good accuracy [6]. Statistical models with assumptions on process effects were used to define, whether a direct-to-center approach might be beneficial for patients that were detected as “likely LVO” with a prehospital scale [7]. Some of these models suggest such a benefit, particularly in urban regions [7–9]. However, a direct-to-center approach on the basis of a scale trades one recanalizing treatment (delaying IVT) against another one (accelerating EVT). Only one randomized controlled trial (RCT) investigating clinical outcome has been conducted so far looking into the direct-to-center approach. The RACE-CAT trial randomized patients with a RACE score of ≥ 5pts into prehospital transfer to a comprehensive stroke center (CSC) versus transfer to the closest IVT-capable center (and subsequent potential interhospital transfer to a CSC). Clinical outcome was not improved by the direct-to-center approach in ischemic stroke patients. And patients with hemorraghic stroke even seemed to come out worse [10]. Therefore, further trials on clinical outcome are mandatory to investigate the utility of prehospital scales with regard to outcome before this may translate into a new strategy of prehospital routing. Guidelines therefore suggest transporting a patient suspected of having a stroke to the closest IVT-capable stroke center irrespective of the severity of the stroke. Regional and situative particularities may nevertheless support the use of prehospital severity scales to help with transport decisions. It is of utmost importance that secondary transport must not be delayed in patients with LVO who receive IVT in a primary center. Networks and SOPs between centers can help to solve this problem.
5. A different approach is that of MSUs. An ambulance is equipped with a CT-scanner that provides non-contrast CT (NCCT) and CT-Angiography (CTA) imaging as well as a point of care (PoC) laboratory. In this setting, patients can be reliably identified as stroke patients with or without LVO. Then, IVT can be given on scene and in case of LVO, a direct transfer to an EVT-capable center can be initiated. Several trials showed that time delay until IVT is reduced and clinical outcome is significantly improved [11–13]. Therefore, in areas in which stroke volume is high (e.g. metropolitan areas), MSU should be considered, if the means for the setup are available.
6. After admission to the primary (and possibly definitive) hospital, the patient needs to receive a rapid yet thorough ER workup. After checking and possibly securing vital functions, taking the temperature and initiating monitoring [blood pressure (BP), EKG and heart rate, respiratory rate, oxgen saturation (SpO_2_)], this should involve a set of laboratory tests including coagulation parameters, possibly including Xa acivity tests. The latter is of particular importance if the patient is taking oral anticoagulants. While these still constitute an official contraindication against IVT (details on this are beyond the scope of this article) they do not seem to be problematic with regard to off-label IVT if no effective blood levels are seen, even following reversal or not [14]. Again, local concepts of ER workups may differ. Stroke team training by simulation workshops [15], standardization by “Code Stroke” or fast-track pathways such as the “One-Stop-Shop“ concept [16] and other approaches to streamline the process [17, 18] are ways to speed up stroke logistics in the ER and cut down treatment times. After bedside diagnostics, the patient needs to be prepared for brain imaging and further treatment by at best two peripheral venous lines. Oxygen by mask should only be delivered to patients who are hypoxic, but not routinely. Preferrably, workup elements are combined in one place, such as the CT— or angiography suite, a concept that has proved to safe valuable time [19].
7. It is of very high importance that the patient receives rapid and adequate cerebral imaging. In the past and still customary in many primary hospitals today, NCCT combined with CTA is still the imaging method of choice for the primary assessment of any patient with clinical criteria qualifying for recanalization treatment. In Germany, it is now a prerequisite for any SU to be able to do CT- or MR-Angiography (MRA) 24/7. CT-Perfusion (CTP) can be used as an advanced imaging method useful in special scenarios like prolonged or unclear time windows of stroke onset (see also 10.). Above all, imaging has to rule out ICH or readily appreciable „stroke mimics“ (such as migraine, postictal state or subarachnoid hemorrhage) or else confirm the suspicion of AIS. Some stroke mimics may not present with abnormalities on imaging, such as epileptic seizures, however only few patients with presumed stroke are ultimately not diagnosed with a stroke and the complication rate associated with IVT in stroke mimics is very low [20]. NCCT can be used to assess infarct volume with standardized scores such as the Alberta Stroke Program Early CT Score (ASPECTS) [21]. An important result of primary imaging in patients with severe AIS is advanced infarct. The guidelines of the European stroke organization (ESO) recommend EVT for patients with ASPECTS above 5 or infarct core below 70 ml [22]. Although there remains controversy as to the indication of EVT in large infarcts, and advanced imaging like CTP which may be needed to better identify infarct core and salvageable tissue to support the indication for EVT, the evidence for beneficiary effects on patient outcome after EVT for LVO in larger infarct is growing. Recent RCTs showed that even in patients with large infarcts (dASPECTS 3–5) EVT resulted in better functional outcomes than medical care alone [23–27] and might soon change current guideline recommendations. However, in some of these trials, rates of symptomatic ICH were numerically and of asymptomatic ICH significantly higher in thrombectomized patients, and there was no effect on rates of decompressive hemicraniectomy. Although we consider CT + CTA the method of choice for diagnosis of stroke and LVO, MRI including MRA is a feasible alternative in some hospitals and may facilitate the distinction between stroke and some stroke mimics as well as identify patients with mismatch between infarct core and salvageable brain tissue (see also 13.).
8. Patients in which imaging has shown ICH need to be managed according to current ICH guidelines, with the acute main measures of reducing BP and possibly normalizing coagulation. They need to the managed in a SU or -if space-occupying- a NCCU. Those with stroke mimics such as migraine or seizures should certainly be managed according to these conditions. Some stroke mimics may demand advanced imaging to be diagnosed. However, if doubt remains and the suspicion of AIS cannot be ruled out, IVT should still be aimed for and appears to be quite safe (see also 7.). Patients with advanced AIS, i.e. those with no signs of salvageable brain tissue, either need to be directly admitted to the SU or the NCCU, depending on their clinical state, without further attempts of recanalization (see also 7.). Admission to the NCCU is recommended in patients who are compromised with regard to their vital functions as often in brainstem stroke (including severe dysphagia and need of artificial airway protection), have space-occupying infarcts, are likely to need neurosurgical interventions such as decompression, or have other reasons to receive critical care (see also 19.).
9. In patients with unclear or prolonged time window (exceeding 4.5 h in patients planned for IVT or 6 h for EVT) it is reasonable to exclude larger infarcts or those without salvageable brain tissue from recanalization therapies. Mostly this is done using perfusion imaging and comparing different criteria from this imaging (e.g. cerebral blood flow (CBF) and time to peak (TTP) in CT-Perfusion, or DWI and TTP in MR-imaging). Still, it is matter of debate which are the best criteria, and no strict recommendation can be given. The trial DEFUSE-3 used a target mismatch profile on CT or MRI between infarct core (< 70 ml) and critical hypoperfusion (Tmax > 6 s; > 15 ml and ratio > 1.8) [28], whereas the trial DAWN used a clinical-imaging mismatch (age 18–79 years: National Institutes of Health Stroke Scale (NIHSS) score ≥ 10 and infarct core 0-30 ml; NIHSS ≥ 20 and infarct core 31-51 ml; age ≥ 80 years: NIHSS ≥ 10 and infarct core 0–20 ml) [29]. Whether this can be translated to a mismatch between severe clinical symptoms and minor early infarct signs on CT [30], is also matter of debate and needs further evaluation before it can be used in clinical routine. The recent MR CLEAN-LATE trial used collateral flow to indicate EVT in a time window of 6-24 h, again with positive results [31]. Another mismatch concept was evaluated in the WAKE-UP trial. Using standard MRI for patients with unclear time of onset but likely symptoms duration of less than 4.5 h, a short time-window was supposed in those where the infarcts was visible on DWI but not yet in the FLAIR-sequence. In patients with such a DWI-FLAIR-mismatch IVT was superior to standard care [32].
10. An approach to achieve rapid EVT in rural areas may be that of a mobile intervention team. In this scenario, EMS transports the suspected stroke patients to the closest primary hospital with a stroke unit (SU) and an (often cardiologic) angiography, where acute stroke diagnosis is performed. A team of interventionists is provided by an EVT-capable center and is driven by car or flown via helicopter to the primary hospital if the patient is in need of EVT, in order to perform the intervention onsite. The approach with a car has shown to reduce time delay until EVT [33–35]. The flying intervention team via helicopter was compared to secondary transfer in a controlled intervention study [36]. Time until thrombectomy was reduced by 90 min and the study showed equivalent rates of technical success and complications in both groups. Generalizability of this system of care has yet to be shown. These different access concepts are increasingly provided within dedicated stroke networks, usually organized as hub-and-spoke systems employing telemedicine.
11. Whether IVT and EVT should be combined in patients suitable for both modalities was evaluated in several trials. The recent update of the German acute treatment guideline recommends to combine IVT and EVT in patients within a 4.5 h time-window, if no contraindication is present. For the time-window between 4.5 and 9 h there is very limited evidence for the combined therapy. The German guideline committee therefore recommends that an EVT alone might be considered if possible without delay. And in patients treated with EVT in a time-window beyond 9 h no IVT is recommended [1]. The ESO recommends to use alteplase for bridging in wake-up patients applying the EXTEND and WAKE-UP criteria [3].
12. Most evidence for the efficacy of EVT comes from studies in patients with occlusion of a vessel in the anterior circulation. In patients with basilar artery occlusion (BAO), IVT with alteplase was similarly effective to intra-arterial lysis therapy in the BASICS registry [37]. Two RCTs comparing EVT with best medical treatment (including IVT) had not shown a convincing benefit of EVT, but the studies were small and had high cross-over rates [38, 39]. The recently published ATTENTION trial showed almost a doubling of the proportion of patients with good clinical outcome (mRS 0–3) after EVT [40]. Around a third of the patients were also treated with IVT, and subgroup analysis showed a smaller benefit of EVT in those patients. Similarly, the recent BAOCHE trial demonstrated better functional outcome for EVT in a 6–24 h window than best medical treatment alone, despite more complications and hemorrhages [9]. Therefore, we currently do not see much difference in recanalizing therapy for patients with BAO compared to patients with supratentorial stroke, with the exception that a penumbral mismatch concept is less established in the vertebrobasilar territory. Although there is no evidence from RCTs regarding the role of bridging IVT in posterior circulation LVO (BA, PCA), at least retrospective studies show a trend toward better functional outcome and reduced mortality in patients receiving it. In summary, we advocate IVT before EVT in posterior circulation LVO whenever possible.
13. For patients with unclear time-window the recanalization strategy should be based on multimodal imaging. In patients with a severe deficit (e.g. NIHSS ≥ 6, based on DEFUSE-3 [28]) CTP-based mismatch could be used to indicate EVT, in patients with less severe symptoms DWI-FLAIR mismatch can be used to indicate IVT based on the WAKE-Up trial [32]. Combination-therapy would be very rare in those patients.
14. Official contraindications against thrombolysis are listed in Table 1. However, most of these do not have pathophysiologic backgrounds and showed little clinical relevance in large registries. They are therefore regarded as only relative contraindications or even “ignored” in many comprehensive stroke centers. Medicolegally, however, treatment has to be justified if any official contraindication is present. Therefore, a thorough documentation of the contraindication and the reason why IVT was initiated despite one or more being present, is recommended.
15. IVT is the administration of alteplase at a dose of 0.9 mg/kg body weight (bw) over a period of one hour. Treatment with a reduced dose of 0.6 mg is not recommended [1]. Tenecteplase is another thrombolytic substance with higher fibrin specificity, which can be administered as a single bolus. Several trials have been or are being perfomed, comparing alteplase and tenecteplase in AIS patients. The updated German stroke guideline concluded that it is reasonable using tenecteplase (0.25 mg/kg bw) as an alternative to alteplase, especially in patients with large vessel occlusion, in which also an EVT is planned [1]. However, in Europe tenecteplase has no label for stroke treatment yet and is available only in 50 mg package size, which is twice as much as needed for stroke treatment. Patients who are treated with IVT need to be observed on a SU or an ICU. Strict monitoring is recommended, for example of the (systolic) blood pressure which needs to be lowered below 180 mmHg if necessary in patients receiving IVT and is probably reasonable to be kept between 140 and 160 mmHg. Patients need to be screened for bleeding complications, including intracranial hemorrhage. A rare complication of alteplase is an oropharyngeal edema; IVT has to be stopped immediately if this occurs and antihistamines and corticosteroids be given. Patients who are treated with IVT need to be observed on a SU or an ICU. Strict monitoring is recommended, for example of the (systolic) blood pressure which needs to be lowered below 180 mmHg if necessary in patients receiving IVT and is probably reasonable to be kept between 140 and 160 mmHg. Patients need to be screened for bleeding complications, including intracranial hemorrhage. A rare complication of alteplase is an oropharyngeal edema; IVT has to be stopped immediately if this occurs and antihistamines and corticosteroids be given. It has to be emphasized that IVT certainly is also indicated in patients with measurable neurological deficit and -respecting the contraindications (Table 1)- regardless of the presence of LVO.
16. When EVT is indicated, a (certified) neurointerventionist should perform the procedure immediately or as soon as possible. Nowadays combined techniques for EVT in LVO of the anterior circulation are preferred as retrospective studies have shown higher first pass rates, shorter procedure times and higher rates of complete reperfusion [41]. These techniques encompass the use of balloon guide catheters, aspiration catheters and stent retrievers. Nevertheless, alternative established techniques like direct aspiration or (± balloon) guide catheter (± aspiration catheter) + stent retriever are effective and safe as well [42, 43]. The ideal technical approach to EVT taking into account access vessel tortuosity, vessel occlusion site and assumed clot composition is still a matter of debate. EVT of anterior circulation LVO has shown to be beneficial for patient outcome independent from the applied technique, so the local availability of material and devices should be considered, and techniques adapted to it. Full recanalization in terms of expanded Treatment In Cerebral Infarction (eTICI) grades 2c-3 should be aimed for as increasing TICI grades are linked with better outcomes [42]. When standard techniques fail, rescue strategies can be applied in EVT. Accessing the target vessel can sometimes be impossible through the standard femoral or radial approach. Then direct carotid puncture (DCP) can be a manageable alternative. The decision to perform a DCP should not be delayed after it becomes clear that a standard approach is prone to failure as time to recanalization is on average more than two hours longer when DCP was performed [44]. Of note is the substantially higher rate of complications attributed to DCP in almost 20% of cases like cervical hematoma, complete occlusion and pseudoaneurysm. When the clot itself is refractory to retrieval, several rescue maneuvers can be considered such as fast stent retrieval [45], double stent retriever technique [46], stent retriever technique [47, 48] or permanent intracranial stenting [49, 50]. In posterior circulation LVO, atherosclerotic disease as the underlying stroke cause is more common than in the anterior circulation [51]. Therefore, rescue strategies like intracranial stenting are more often in need. To aim for rapid application of recanalizing therapies, local protocols should contain time beams with targets, such as: (Door → CT ≤ 10 min; Door → IVT ≤ 30 min; Door → Groin ≤ 60 min).
17. Device- or procedure-related complications occur in 4% to 29% of EVT procedures [52]. Extracranial complications mainly comprise access site related hemorrhage or dissection. Intracranial complications subdivide into potentially ischemic complications, including vasospasm, dissection, emboli to new or distal territories in reference to the initially occluded vessel, and potentially hemorrhagic complications like vessel perforation or vessel stretching with avulsion of perforating arteries with subsequent subarachnoid hemorrhage [52]. It has to be part of the neurointerventionist’s diligence to learn about strategies for prevention and management of these complications. Furthermore, especially in stroke patients presenting in prolonged time-windows or with advanced infarction we tend to expect a higher rate of hemorrhagic transformations and symptomatic intracranial hemorrhage (SIH) after EVT. However, recent studies showed that EVT compared to best medical treatment is not associated with a higher risk for SIH [23, 53] in well-selected patients.
18. Managing the patient before, during and after thrombectomy (coined “periinterventional management”) involves preparation (gown, peripheral lines, bladder catheter or alternative, positioning), monitoring (BP, EKG, SpO2, etc.) and neuroanesthetic treatment (volume, catecholamines, possibly intubation and ventilation, etc.) [54]. Considerable controversy exists around the topics sedation and blood pressure, while other potentially relevant aspects (such as temperature, glucose, neuromonitoring, etc.) have not been sufficiently investigated. In addition to implications to steer blood pressure according to whether the patient has received IVT, the course and result of EVT seem to be associated with certain blood pressure trajectories, outcomes and treatment implications. The latter are still being elucidated. According to one study, a sustained mean arterial blood pressure *during* the intervention lower than 70 mmHg or higher than 90 mmHg appears deleterious [55]. A higher SBP *after* EVT is associated with more symptomatic ICH and worse functional outcome [56, 57] or death [58]. However, a RCT investigating intensive SBP lowering to < 120 mmHg after EVT found worse functional outcome in that group [59]. At present, it appears reasonable to keep SBP between 140 and 180 mmHg after (successful) EVT; more prospective research on BP is warranted. Quite likely, optimal BP goals during and after EVT are very individual. A tool to taylor BP after EVT successfully may be transcranial Doppler/Duplex (TCD) sonography [60, 61]. As to sedation, it is customary to do EVT with patients intubated and under general anesthesia (GA) in many places while in other centers procedural sedation (PS) is preferred, meaning no intubation and only light sedation and/or analgesia. GA may have the advantages of an immobile patient not disturbing the intervention, a secured airway and more patient comfort, PS may have the advantages of no time delay, no circulatory compromise and easier post-interventional logistics. Although many retrospective studies including post-hoc analyses of endovascular landmark trials have suggested worse outcomes with GA and therefore discouraged it [62], these disadvantages could not be confirmed in randomized trials, which were either neutral or supportive regarding GA [63, 64]. Current guidelines do not recommend one sedation strategy over the other -yet- but rather locally, individually based approaches, the application of protocols as well as dedicated anesthesiologic or neurocritical periinterventional care teams [1, 22].
19. All patients with AIS need to be admitted to a SU. Further management there involves monitoring and stabilization of cardiopulmonary function, neurologic assessment, initiation of early secondary stroke prevention, physiotherapy/speech and swallow therapy/occupational therapy, and prevention of complications such as thrombosis and pulmonary embolism, delirium, pneumonia or other infections. After EVT, vital parameters and other factors (e.g. targeting glucose levels at 140–180 mmHg, normothermia, groin check for aneurysm or hematoma, NIHSS, neurovascular ultrasound, control imaging, etc.) need extra attention. Many patients cool down in the aniography suite (which can be avoided by a warming mattress). Although some consider that a potential protective side effect (and set out to investigate hypothermia during EVT), it may cause disadvantages such as delayed extubation or delirium. At present, we therefore advocate to keep normothermia during and after EVT. These patients may still deteriorate clinically, for instance if they acquire such complications or if their stroke progresses, e.g. by edema and space-occupying effect in the scull. Patients may then have to be transferred to a NCCU. Reasons to transfer patients there directly from the angiography suite include failed EVT or inability to extubate early. At the NCCU, ventilation and airway measures, circulatory support, neuromonitoring, and possibly pre- and post-operative care for decompressive craniectomy are predominant [65].

**Table 1:** Official indications and contraindications for Alteplase in acute stroke treatment

*Indications*
Diagnosis of ischemic stroke causing a measurable disabling neurologic deficit
Onset of symptoms < 4.5 h before beginning treatment
Age ≥ 16 years
*Clear contraindications*
Severe head trauma in previous 3 months
Symptoms suggestive of subarachnoid hemorrhage
Previous ICH
Intracranial/spinal surgery in previous 3 months
Intracerebral neoplasm
Infective endocarditis
Aortic arch dissection
Elevated blood pressure (systolic > 185 mm Hg or diastolic > 110 mm Hg) that cannot be lowered safely
Active internal bleeding
Acute bleeding diathesis, including but not limited to:
Platelet count < 100,000/mm^3^
Heparin received within 48 h with an elevated aPTT (> 40 s)
Current use of treatment doses of low-molecular-weight heparin within the previous 24 h (not applicable to DVT prophylactic dosages of low-molecular-weight heparin)
Current use of anticoagulant with INR > 1.7 or PT > 15 s
Current use of direct thrombin inhibitors or direct factor Xa inhibitors
CT demonstrates infarction (hypodensity) > 1/3 cerebral hemisphere
CT demonstrates an acute ICH
*Relative contraindications*
Mild and nondisabling or rapidly improving stroke symptoms
Very severe neurologic deficits (NIHSS score > 25) within the 3- to 4.5 h window
Pregnancy
Seizure at onset (consider Alteplase if neurologic deficits are thought to be caused by a stroke)
Arterial puncture at non-compressible site in previous 7 days
Untreated intracranial arteriovenous malformation
Untreated giant intracranial aneurysm
Recent major surgery or serious trauma (within previous 14 days)
Recent gastrointestinal or urinary tract hemorrhage (within previous 21 days)
Ischemic stroke within previous 3 months
Recent ST-elevation acute myocardial infarction (within previous 3 months)
Blood glucose concentration < 50 mg/dL (2.7 mmol/L)

## Conclusion

Providing optimal therapy to the individual patient with severe acute ischemic stroke may be supported by the systematic application of the SOP suggested above which can only be a template to be adapted to local conditions, of course. Although based for the great part on current guidelines and landmark studies, scientific progress in this area of stroke care is rapid and likely to change soon, hence vigilance for updates is mandatory. The authors wish to emphasize, that although spectacular results can be achieved in individual patients many hours after stroke onset today by the combination of selective imaging, thrombolysis and thrombectomy, time delay must still be avoided by all means. And, since stroke thrombectomy may only be adequate for 15–30% of patients with ischemic stroke, the rapid application of indicated thrombolysis to stroke patients remains the most important pillar of acute recanalization worldwide.

## Data Availability

All source literature for this SOP article were available to all authors. Making data available to others is not applicable to this article format.

## Abbreviations

ABC: Airway breathing circulation
ABCDE: Airway breathing circulation disability exposure
AIS: Acute ischemic stroke
ASPECTS: Alberta stroke program early CT score
BAO: Basilar artery occlusion
BGA: Blood gas analysis
BP: Blood pressure
Bw: Body weight
CBC: Complete blood count
CBF: Cerebral blood flow
CPSS: Cincinnati prehospital stroke scale
CSC: Comprehensive stroke center
CTA: Computed tomography angiography
CTP: Computed tomography perfusion
DCP: Direct carotid puncture
DWI: Diffusion weighted imaging
eTICI: Expanded treatment in cerebral infarction (grade)
FLAIR: Fluid-attentuated inversion recovery
GA: General anesthesia
EMS: Emergency medical service
ER: Emergency room
ESO: European stroke organisation
EVT: Endovascular (stroke) treatment
FAST: Face-arm-speech-time (test)
ICU: Intensive care unit
ICH: Intracerebral hemorrhage
IVT: Intravenous thrombolysis
LAMS: Los Angeles motor scale
LVO: Large vessel occlusion
MRA: Magnetic resonance angiography
MSU: Mobile stroke unit
NIHSS: National Institutes of Health Stroke Scale
NCCT: Non-contrast computed tomography
NCCU: Neurocritical care unit
PoC: Point of care
RACE: Rapid arterial occlusion evaluation (scale)
RCT: Randomized controlled trial
SIH: Symptomatic intracranial hemorrhage
SBP: Systolic blood pressure
SpO_2_: Blood oxygen saturation
SU: Stroke unit
TICI: Thrombolysis in cerebral infarction (score)
TSH: Thyroid-stimulating hormone
TTP: Time to peak

## References

[1] Ringleb, P., Köhrmann, M., Hametner, C., et al. (2022). Akuttherapie des ischämischen Hirninfarktes, S2- Leitlinie, Version 5.122.11.2022. https://www.awmf.org/leitlinien/detail/ll/030-046.html (accessed 22 Nov 2022).

[2] Powers WJ, Rabinstein AA, Ackerson T (2019). Guidelines for the early management of patients with acute ischemic stroke: 2019 update to the 2018 guidelines for the early management of acute ischemic stroke: A guideline for healthcare professionals from the American Heart Association/American Stroke Association. Stroke.

[3] Berge E, Whiteley W, Audebert H (2021). European Stroke Organisation (ESO) guidelines on intravenous thrombolysis for acute ischaemic stroke. European Stroke Journal.

[4] Katsanos AH, Psychogios K, Turc G (2022). Off-label use of tenecteplase for the treatment of acute ischemic stroke: A systematic review and meta-analysis. JAMA Network Open.

[5] Zhelev Z, Walker G, Henschke N, Fridhandler J, Yip S (2019). Prehospital stroke scales as screening tools for early identification of stroke and transient ischemic attack. Cochrane Database Systematic Review.

[6] Duvekot MHC, Venema E, Rozeman AD (2021). Comparison of eight prehospital stroke scales to detect intracranial large-vessel occlusion in suspected stroke (PRESTO): A prospective observational study. Lancet Neurology.

[7] Venema E, Lingsma HF, Chalos V (2019). Personalized prehospital triage in acute ischemic stroke. Stroke.

[8] Zhao H, Smith K, Bernard S (2021). Utility of severity-based prehospital triage for endovascular thrombectomy: ACT-FAST validation study. Stroke.

[9] Jovin TG, Li C, Wu L (2022). Trial of thrombectomy 6 to 24 hours after stroke due to basilar-artery occlusion. N Engla J Med.

[10] de Perez ON (2022). Effect of direct transportation to thrombectomy-capable center vs local stroke center on neurological outcomes in patients with suspected large-vessel occlusion stroke in nonurban Areas: The RACECAT randomized clinical trial. JAMA.

[11] Grotta JC, Yamal JM, Parker SA (2021). Prospective, multicenter, controlled trial of mobile stroke units. New England Journal of Medicine.

[12] Ebinger M, Winter B, Wendt M (2014). Effect of the use of ambulance-based thrombolysis on time to thrombolysis in acute ischemic stroke: A randomized clinical trial. JAMA.

[13] Ebinger M, Siegerink B, Kunz A (2021). Association between dispatch of mobile stroke units and functional outcomes among patients with acute ischemic stroke in Berlin. JAMA.

[14] Meinel TR, Wilson D, Gensicke H (2023). Intravenous thrombolysis in patients with ischemic stroke and recent ingestion of direct oral anticoagulants. JAMA Neurology.

[15] Tahtali D, Bohmann F, Kurka N (2017). Implementation of stroke teams and simulation training shortened process times in a regional stroke network-A network-wide prospective trial. PLoS ONE.

[16] Psychogios M-N, Maier IL, Tsogkas I, Hesse AC, Brehm A, Behme D, Schnieder M, Schregel K, Papageorgiou I, Liebeskind DS, Goyal M, Bähr M, Knauth M, Liman J (2019). One-stop management of 230 consecutive acute stroke patients: Report of procedural times and clinical outcome. Journal of Clinical Medicine.

[17] Meretoja A, Weir L, Ugalde M (2013). Helsinki model cut stroke thrombolysis delays to 25 minutes in Melbourne in only 4 months. Neurology.

[18] Schregel K, Behme D, Tsogkas I (2016). Effects of workflow optimization in endovascularly treated stroke patients - a pre-post effectiveness study. PLoS ONE.

[19] Wu TY, Coleman E, Wright SL (2018). Helsinki stroke model is transferrable with "real-world" resources and reduced stroke thrombolysis delay to 34 min in christchurch. Frontiers in Neurology.

[20] Ali-Ahmed F, Federspiel JJ, Liang L (2019). Intravenous Tissue plasminogen activator in stroke mimics. Circulation. Cardiovascular Quality and Outcomes.

[21] Barber L, Barrett R, Lichtwark G (2011). Validity and reliability of a simple ultrasound approach to measure medial gastrocnemius muscle length. J Anatomy.

[22] Turc G, Bhogal P, Fischer U (2019). European stroke organisation (ESO) - European Society For Minimally Invasive Neurological Therapy (ESMINT) Guidelines on Mechanical Thrombectomy in Acute Ischaemic StrokeEndorsed by Stroke Alliance for Europe (SAFE). European Stroke Journal.

[23] Sarraj A, Hassan AE, Abraham MG (2023). Trial of endovascular thrombectomy for large Ischemic Strokes. New England Journal of Medicine.

[24] Huo X, Ma G, Tong X (2023). Trial of endovascular therapy for acute ischemic stroke with large infarct. New England Journal of Medicine.

[25] Yoshimura S, Sakai N, Yamagami H (2022). Endovascular therapy for acute stroke with a large ischemic region. New England Journal of Medicine.

[26] Huo X, Ma G, Tong X (2023). Trial of endovascular therapy for acute ischemic stroke with large infarct. New England Journal of Medicine.

[27] Sarraj A, Hassan AE, Abraham MG (2023). Trial of endovascular thrombectomy for large ischemic strokes. New England Journal of Medicine.

[28] Albers GW, Marks MP, Kemp S (2018). Thrombectomy for stroke at 6 to 16 hours with selection by perfusion imaging. New England Journal of Medicine.

[29] Nogueira RG, Jadhav AP, Haussen DC (2017). Thrombectomy 6 to 24 hours after stroke with a mismatch between deficit and infarct. New England Journal of Medicine.

[30] Sykora M, Kellert L, Michel P (2020). Thrombolysis in stroke with unknown onset based on non-contrast computerized tomography (TRUST CT). Journal of the American Heart Association.

[31] Olthuis SGH, Pirson FAV, Pinckaers FME (2023). Endovascular treatment versus no endovascular treatment after 6–24 h in patients with ischaemic stroke and collateral flow on CT angiography (MR CLEAN-LATE) in the Netherlands: A multicentre, open-label, blinded-endpoint, randomised, controlled, phase 3 trial. Lancet.

[32] Thomalla G, Simonsen CZ, Boutitie F (2018). MRI-guided thrombolysis for stroke with unknown time of onset. New England Journal of Medicine.

[33] Brekenfeld C, Goebell E, Schmidt H (2018). 'Drip-and-drive': Shipping the neurointerventionalist to provide mechanical thrombectomy in primary stroke centers. J Neurointerv Surg.

[34] Morey JR, Oxley TJ, Wei D (2020). Mobile interventional stroke team model improves early outcomes in large vessel occlusion stroke: The NYC MIST trial. Stroke.

[35] Seker F, Mohlenbruch MA, Nagel S (2018). Clinical results of a new concept of neurothrombectomy coverage at a remote hospital-"drive the doctor". International Journal of Stroke.

[36] Hubert GJ, Hubert ND, Maegerlein C (2022). Association between use of a flying intervention team vs patient interhospital transfer and time to endovascular thrombectomy among patients with acute ischemic stroke in Nonurban Germany. JAMA.

[37] Schonewille WJ, Wijman CA, Michel P (2009). Treatment and outcomes of acute basilar artery occlusion in the Basilar Artery International Cooperation Study (BASICS): A prospective registry study. Lancet Neurology.

[38] Liu X, Dai Q, Ye R (2020). Endovascular treatment versus standard medical treatment for vertebrobasilar artery occlusion (BEST): An open-label, randomised controlled trial. Lancet Neurology.

[39] Langezaal LCM, van der Hoeven E, Mont'Alverne FJA (2021). Endovascular therapy for stroke due to basilar-artery occlusion. New England Journal of Medicine.

[40] Tao C, Nogueira RG, Zhu Y (2022). Trial of endovascular treatment of acute basilar-artery occlusion. New England Journal of Medicine.

[41] Maegerlein C, Berndt MT, Monch S (2020). Further development of combined techniques using stent retrievers, aspiration catheters and BGC : The PROTECT(PLUS) technique. Clinical Neuroradiology.

[42] Lapergue B, Blanc R, Costalat V (2021). Effect of thrombectomy with combined contact aspiration and stent retriever vs stent retriever alone on revascularization in patients with acute ischemic stroke and large vessel occlusion: the ASTER2 randomized clinical trial. JAMA.

[43] Turk AS, Siddiqui A, Fifi JT (2019). Aspiration thrombectomy versus stent retriever thrombectomy as first-line approach for large vessel occlusion (COMPASS): A multicentre, randomised, open label, blinded outcome, non-inferiority trial. Lancet.

[44] Dumas V, Kaesmacher J, Ognard J (2022). Carotid artery direct access for mechanical thrombectomy: The carotid artery puncture evaluation (CARE) study. J Neurointerv Surg.

[45] Soize S, Eymard JB, Cheikh-Rouhou S (2021). Fast stent retrieval during mechanical thrombectomy improves recanalization in patients with the negative susceptibility vessel sign. AJNR. American Journal of Neuroradiology.

[46] Imahori T, Miura S, Sugihara M, Mizobe T, Aihara H, Kohmura E (2020). Double stent retriever (SR) technique: A novel mechanical thrombectomy technique to facilitate the device-clot interaction for refractory acute cerebral large vessel occlusions. World Neurosurg.

[47] Aydin K, Barburoglu M, Oztop Cakmak O, Yesilot N, Vanli ENY, Akpek S (2019). Crossing Y-Solitaire thrombectomy as a rescue treatment for refractory acute occlusions of the middle cerebral artery. J Neurointerv Surg.

[48] Moreu M, Perez-Garcia C, Gomez-Escalonilla C, Rosati S (2020). Dual SAVE technique for mechanical thrombectomy rescue on MCA bifurcation clots. J Neurointerv Surg.

[49] Chang Y, Kim BM, Bang OY (2018). Rescue stenting for failed mechanical thrombectomy in acute ischemic stroke: A multicenter experience. Stroke.

[50] Maingard J, Phan K, Lamanna A (2019). Rescue intracranial stenting after failed mechanical thrombectomy for acute ischemic stroke: A systematic review and meta-analysis. World Neurosurg.

[51] Mutke, M. A., Potreck, A., Schmitt, N., Seker, F., Ringleb, P. A., Nagel, S., Möhlenbruch, M. A., Bendszus, M., Weyland, C. S., & Jesser, J. (2022). Exact basilar artery occlusion location indicates stroke etiology and recanalization success in patients eligible for endovascular stroke treatment. *Clinical Neuroradiology*. 10.1007/s00062-022-01236-0 PMC10219858 36459175

[52] Pilgram-Pastor SM, Piechowiak EI, Dobrocky T (2021). Stroke thrombectomy complication management. J Neurointerv Surg.

[53] Nogueira RG, Jadhav AP, Haussen DC (2018). Thrombectomy 6 to 24 hours after stroke with a mismatch between deficit and infarct. New England Journal of Medicine.

[54] Bosel J (2016). Intensive care management of the endovascular stroke patient. Seminars in Neurology.

[55] Rasmussen M, Schonenberger S, Henden PL (2020). Blood pressure thresholds and neurologic outcomes after endovascular therapy for acute ischemic stroke: An analysis of individual patient data from 3 randomized clinical trials. JAMA Neurology.

[56] Petersen NH, Kodali S, Meng C (2022). Blood pressure trajectory groups and outcome after endovascular thrombectomy: A multicenter study. Stroke.

[57] Samuels N, van de Graaf RA, van den Berg CAL (2021). Blood pressure in the first 6 hours following endovascular treatment for ischemic stroke is associated with outcome. Stroke.

[58] Katsanos AH, Malhotra K, Ahmed N (2022). Blood pressure after endovascular thrombectomy and outcomes in patients with acute ischemic stroke: An individual patient data meta-analysis. Neurology.

[59] Yang P, Song L, Zhang Y (2022). Intensive blood pressure control after endovascular thrombectomy for acute ischaemic stroke (ENCHANTED2/MT): A multicentre, open-label, blinded-endpoint, randomised controlled trial. Lancet.

[60] Kneihsl M, Niederkorn K, Deutschmann H (2018). Increased middle cerebral artery mean blood flow velocity index after stroke thrombectomy indicates increased risk for intracranial hemorrhage. J Neurointerv Surg.

[61] Baracchini C, Farina F, Palmieri A (2019). Early hemodynamic predictors of good outcome and reperfusion injury after endovascular treatment. Neurology.

[62] Campbell BCV, van Zwam WH, Goyal M (2018). Effect of general anaesthesia on functional outcome in patients with anterior circulation ischaemic stroke having endovascular thrombectomy versus standard care: A meta-analysis of individual patient data. Lancet Neurology.

[63] Schonenberger S, Henden PL, Simonsen CZ (2019). Association of general anesthesia vs procedural sedation with functional outcome among patients with acute ischemic stroke undergoing thrombectomy: A systematic review and meta-analysis. JAMA.

[64] Campbell D, Butler E, Campbell RB, Ho J, Barber PA (2023). General anesthesia compared to Non-GA in endovascular thrombectomy for ischemic stroke: A systematic review and meta-analysis of randomized controlled trials. Neurology.

[65] Chang CWJ, Provencio JJ, Shah S (2021). Neurological critical care: The evolution of cerebrovascular critical care. Critical Care Medicine.

